# Multilocus genotypes and broad host-range of *Enterocytozoon bieneusi* in captive wildlife at zoological gardens in China

**DOI:** 10.1186/s13071-016-1668-1

**Published:** 2016-07-08

**Authors:** Wei Li, Lei Deng, Xingming Yu, Zhijun Zhong, Qiang Wang, Xuehan Liu, Lili Niu, Na Xie, Jiabo Deng, Shuangshuang Lei, Liqin Wang, Chao Gong, Ziyao Zhou, Yanchun Hu, Hualin Fu, Huailiang Xu, Yi Geng, Guangneng Peng

**Affiliations:** The Key Laboratory of Animal Disease and Human Health of Sichuan Province, College of Veterinary Medicine, Sichuan Agricultural University, Chengdu, Sichuan Province 611130 China; The Chengdu Zoo, Institute of Wild Animals, Chengdu, Sichuan Province 625001 China; College of Life Science, Sichuan Agricultural University, Ya’an, 625014 China

**Keywords:** *Enterocytozoon bieneusi*, Wildlife, ITS, MLST, China, Zoological garden

## Abstract

**Background:**

*Enterocytozoon bieneusi* is a common opportunistic pathogen that is widely detected in humans, domestic animals and wildlife, and poses a challenge to public health. The present study was performed to evaluate the prevalence, genotypic diversity and zoonotic potential of *E. bieneusi* among wildlife at Chengdu and Bifengxia zoological gardens in Sichuan Province, China.

**Results:**

Of the 272 fresh fecal samples harvested from 70 captive wildlife species at Chengdu Zoo (*n* = 198) and Bifengxia Zoo (*n* = 74), 21 (10.6 %) and 22 (29.7 %) tested positive for *E. bieneusi* by internal transcribed spacer (ITS) sequencing analysis, respectively. Specifically, genotypes D, Peru 6, CHB1, BEB6, CHS9, SC02 and SC03, and genotypes D, CHB1, SC01 and SC02 were detected in the Chengdu and Bifengxia Zoo samples, respectively. Five known genotypes (D, Peru 6, BEB6, CHS9 and CHB1) and three novel genotypes (SC01, SC02 and SC03) were clustered into the zoonotic group (group 1) and host-adapted group (group 2). Multilocus sequence typing (MLST) analysis targeting three microsatellites (MS1, MS3 and MS7) and one minisatellite (MS4) were successfully sequenced for 37, 33, 35 and 37 specimens, generating 8, 3, 11 and 15 distinct locus types, respectively. Altogether, we identified 27 multilocus genotypes (MLGs) among the *E. bieneusi* isolates by MLST. These data highlight the high genetic diversity of *E. bieneusi* among zoo wildlife.

**Conclusions:**

To our knowledge, this is the first report on the prevalence and genotypic diversity of *E. bieneusi* infections among captive wildlife in zoos in southwest China. Notably, we identified three novel *E. bieneusi* genotypes, as well as six new mammalian hosts (Asian golden cats, Tibetian blue bears, blackbucks, hog deer, Malayan sun bears and brown bears) for this organism. Moreover, the occurrence of zoonotic genotypes suggests that wildlife may act as reservoirs of *E. bieneusi* that can serve as a source of human microsporidiosis. The findings presented here should contribute to the control of zoonotic disease in China.

**Electronic supplementary material:**

The online version of this article (doi:10.1186/s13071-016-1668-1) contains supplementary material, which is available to authorized users.

## Background

Microsporidia, classified as fungi, are the causative agents of microsporidiosis, an important emerging infectious disease [1, 2]. Among the approximately 1,300 microsporidian species identified, *Enterocytozoon bieneusi* is the most frequent cause of microsporidial infections in humans [3]. *Enterocytozoon bieneusi* is an obligate intracellular pathogen that is widely distributed in a variety of animals, including domestic animals and wildlife, and can also be found in water and contaminated food [4–8]. *Enterocytozoon bieneusi* colonizes the epithelium of the small intestine, localizing predominantly within the apical portion of the villus [9]. While microsporidiosis is typically associated with self-limiting diarrhea among healthy individuals, immunocompromised patients, particularly those suffering from AIDS, can develop life-threatening chronic diarrhea [10–13].

Due to the small size of its spores and the uncharacteristic staining properties of this organism, it is difficult to detect *E. bieneusi* by light microscopy [5]. As a result, molecular methods, particularly PCR-based amplification of *E. bieneusi*-specific sequences, are primarily utilized to detect and confirm *E. bieneusi* infections [6]. Currently, due to the high degree of diversity observed among *E. bieneusi* isolates, amplification and sequencing of the ribosomal internal transcribed spacer (ITS) is widely used to identify and genotype these strains [14]. To date, over 200 *E. bieneusi* genotypes, clustered into eight groups (Group 1–8), have been defined [15, 16]. While the strains comprising Group 1 have been isolated from both animals and humans and are generally associated with a major zoonotic potential, those of the other groups are considered host-adapted, as they exhibit a narrow host-range and possess little to no zoonotic potential; however, these organisms remain a potential public health concern [15, 17].

A wide variety of wildlife species are housed at Bifengxia Zoo and Chengdu Zoo. Indeed, Chengdu Zoo is one of the largest zoos in southwest China. Zoo animals are considered domesticated in that they have been separated from their natural habitat. Furthermore, they live under unnatural conditions and in higher densities than those observed in nature [18]. Previous studies have found *Cryptosporidium andersoni* in Bactrian camels and zoonotic *Cryptosporidium* at Bifengxia Zoo [19, 20]. To protect the health of wildlife and to avoid potential public health risks, it is necessary to investigate the occurrence of *E. bieneusi* in captive wild animals. The aim of this study was to examine the prevalence of *E. bieneusi* in various wild animal species in Bifengxia Zoo and Chengdu Zoo, and to genotype the resulting *E. bieneusi* isolates via ITS sequencing and multilocus sequence typing (MLST) analyses. Furthermore, we assessed the zoonotic potential of each *E. bieneusi* strain isolated.

## Methods

### Sample collection and DNA extraction

A total of 272 fecal samples were obtained from wildlife in Chengdu Zoo (*n* = 198) and Bifengxia Zoo (*n* = 74), which are located in Chengdu and Ya’an, respectively, in Sichuan Provence, China, between June 2014 and September 2015. All samples were placed on ice in separate containers, and transported to the laboratory immediately. Prior to use, specimens were stored in 2.5 % potassium dichromate at 4 °C in a refrigerator.

Fecal samples were washed with distilled water and centrifuge at 3,000× *g* for three min. This process was repeated in triplicate. Genomic DNA was then extracted from approximately 200 mg of each semi-purified product using an E.Z.N.A.® Tool DNA Kit (D4015–02; Omega Bio-Tek Inc., Norcross, GA, USA) following the manufacturer’s instructions. DNA samples were stored in 200 μl of the kit Solution Buffer at -20 °C until use.

### Nested PCR amplification and sequencing

*Enterocytozoon bieneusi* was identified by nested PCR amplification of the ITS gene. Multilocus genotyping (MLGs) of ITS-positive specimens was achieved by amplifying three microsatellites (MS1, MS3 and MS7) and one minisatellite (MS4). The primers and amplification conditions for these reactions were as previously described (Table 1) [21, 22]. Each reaction included 12.5 μl 2× Taq PCR Master Mix (KT201-02; Tiangen, Beijing, China), 7.5 μl deionized water (Tiangen), 1 μl 0.1 % bovine serum albumin (BSA; TaKaRa Bio, Shiga, Japan), 2 μl DNA for the primary PCR or primary PCR products 2 μl for the secondary PCR amplification. Secondary PCR products were subjected to 1 % agarose gel electrophoresis and visualized by staining with Golden View. Products of the expected size (392 bp, 675 bp, 537 bp, 885 bp, and 471 bp for ITS, MS1, MS3, MS4 and MS7, respectively) were sent to Invitrogen (Shanghai, China) for two-directional sequencing analysis.

**Table 1:** Primers and annealing temperature for the identification of *Enterocytozoon bieuensi*

Gene locus	Primer sequences (5′–3′)	Annealing temperature (°C)	Expected product size (bp)	Reference
ITS	F1: GATGGTCATAGGGATGAAGAGCTT, R1: AATACAGGATCACTTGGATCCGT	55	410	21
	F2: AGGGATGAAGAGCTTCGGCTCTG, R2: AATATCCCTAATACAGGATCACT	55	392	
MS1	F1: CAAGTTGCAAGTTCAGTGTTTGAA, R1: GATGAATATGCATCCATTGATGTT	58	843	22
	F2: TTGTAAATCGACCAAATGTGCTAT, R2: GGACATAAACCACTAATTAATGTAAC	58	676	
MS3	F1: CAAGCACTGTGGTTACTGTT, R1: AAGTTA GGGCATTTAATAAAATTA	55	702	22
	F2: GTTCAAGTAATTGATACCAGTCT, R2: CTCATTGAATCTAAATGTGTATAA	55	537	
MS4	F1: GCATATCGTCTCATAGGAACA, R1: GTTCATGGTTATTAATTCCAGAA	55	965	22
	F2: CGA AGTGTACTACATGTCTCT, R2: GGACTTTAATAAGTTACCTATAGT	55	885	
MS7	F1: GTTGATCGTCCAGATGGAATT, R1: GACTATCAGTATTACTGATTATAT	55	684	22
	F2: CAATAGTAAAGGAAGATGGTCA, R2: CGTCGCTTTGTTTCATAATCTT	55	471	

### Phylogenetic analyses

All nucleotide sequences obtained in this study were aligned with *E. bieneusi* reference sequences downloaded from the GenBank database using Blast [23] and ClustalX software [24]. Phylogenetic analysis of ITS sequences was performed using Mega software [25], and Maximum Likelihood analysis of the aligned *E. bieneusi* sequences was utilized to support genotype classifications. A total of 1,000 replicates were used for bootstrap analysis.

### Nucleotide sequence GenBank accession numbers

The representative nucleotide sequences for the ITS regions of strains isolated from northern white-cheeked gibbons (*Nomascus leucogenys*), olive baboons (*Papio anubis*), northern raccoons (*Procyon lotor*), golden snub-nosed monkeys (*Rhinopithecus roxellana*), African lions (*Panthera leo*), Asiatic golden cats (*Catopuma temminckii*), giant pandas (*Ailuropoda melanoleuca*), Asiatic black bears (*Ursus thibetanus*), Tibetian blue bears (*Ursus arctos pruinosus*), sika deers (*Cervus nippon*), red pandas (*Ailurus fulgens*), Malayan sun bears (*Helarctos malayanus*), brown bears (*Ursus arctos*), ring-tailed lemurs (*Lemur catta*), alpacas (*Lama pacos*), blackbucks (*Antilope cervicapra*) and hog deers (*Axis porcinus*) were deposited in the GenBank database under the accession numbers KU852462–KU852485 and KX423961. All MS1, MS3, MS4 and MS7 nucleotide sequences obtained in this study were deposited in the GenBank database under accession numbers KU871860–KU871896, KU871897–KU871930, KU871931–KU871965 and KU871966–KU872002, respectively.

## Results

### Prevalence of *E. bieneusi*

In total, 43 of the 272 (15.8 %) animals sampled in this study were infected with *E. bieneusi*. Specifically, 21 of the 198 (10.6 %) and 22 of the 74 (29.7 %) animals sampled from Chengdu and Bifengxia Zoo were *E. bieneusi*-positive, respectively. At Chengdu Zoo, eight (4.0 %), nine (4.5 %), and four (2.0 %) fecal samples from animals of the orders Carnivora, Artiodactyla and Primates were positive for *E. bieneusi*, respectively. At Bifengxia Zoo, 20 (27.0 %) and two (2.7 %) samples from animals of the orders Carnivora and Primates, respectively, contained *E. bieneusi* (Additional file 1: Table S1; Additional file 2: Table S2).

### Genotypes of *E. bieneusi* strains and phylogenetic analysis

In the present study, eight *E. bieneusi* genotypes, comprising five known genotypes (D, Peru 6, BEB6, CHS9, CHB1) and three novel genotypes (SC01, SC02, SC03) were identified by ITS sequencing analysis. Genotype D was detected in an Asiatic golden cat (*n* = 1), African lions (*n* = 2), golden snub-nosed monkeys (*n* = 2), olive baboons (*n* = 2), a northern raccoon (*n* = 1), and a northern white-cheeked gibbon (*n* = 1). Genotype Peru 6 was isolated from a single giant panda (*n* = 1); genotype BEB6 was isolated from a red deer (*n* = 1), hog deer (*n* = 2), an alpaca (*n* = 1), and a sika deer (*n* = 1) and genotype CHS9 was isolated from a blackbuck (*n* = 1) and a hog deer (*n* = 1). Notably, CHB1 was primarily found in Asiatic black bears (*n* = 9), but was also detected in a Malayan sun bear (*n* = 1), a Tibetian blue bear (*n* = 1), a ring-tailed lemur (*n* = 1), a brown bear (*n* = 1) and in red pandas (*n* = 2). Lastly, in regard to the novel genotypes detected, strain SC01 was detected only in Asiatic black bears (*n* = 4), SC02 was isolated from a Tibetian blue bear (*n* = 1), Asiatic black bears (*n* = 3), a sun bear (*n* = 1) and a northern raccoon (*n* = 1), and SC03 was identified in a sika deer (*n* = 1) (Tables 2 and 3).

**Table 2:** List of the mammals in Chengdu Zoo tested positive for *Enterocytozoon bieneusi* in the present study

Common name (scientific name)	No. of positive samples (%)	Genotypes	
		Known	Novel
Order Carnivora	9 (4)	D (3) Peru 6 (1) CHB1 (2)	SC02 (3)
Family Ursidae			
Tibetian blue bear (*Ursus arctos pruinosus*)	2	CHB1 (1)	SC02 (1)
Asiatic black bear (*Ursus thibetanus*)	1		SC02 (1)
Giant panda (*Ailuropoda melanoleuca*)	1	Peru 6 (1)	
Malayan sun bear (*Helarctos malayanus*)	2	CHB1 (1)	SC02 (1)
Family Felidae			
African lion (*Panthera leo*)	2	D (2)	
Asiatic golden cat (*Catopuma temminckii*)	1	D (1)	
Order Artiodactyla	8 (4.5)	BEB6 (5) CHS9 (2)	SC03 (1)
Family Cervidae			
Hog deer (*Axis porcinus*)	3	BEB6 (2) CHS9 (1)	
Red deer (*Cervus elaphus*)	1	BEB6 (1)	
Sika deer (*Cervus nippon*)	2	BEB6 (1)	SC03 (1)
Family Camelidae			
Alpaca (*Lama pacos*)	1	BEB6 (1)	
Family Bovidae			
Blackbuck (*Antilope cervicapra*)	1	CHS9 (1)	
Order Primates	4 (2)	D (4)	
Family Cercopithecidae			
Golden snub-nosed monkey (*Rhinopithecus roxellana*)	2	D (2)	
Olive baboon (*Papio anubis*)	2	D (2)	
Total	21 (10.6)	D (7) Peru 6 (1) CHB1 (2) BEB6 (5) CHS9 (2)	SC02 (3) SC03 (1)

**Table 3:** List of the mammals in Bifengxia Zoo tested positive for *Enterocytozoon bieneusi* in the present study

Common name (scientific name)	No. of positive samples (%)	Genotypes	
		Known	Novel
Order Carnivora	20 (27)	D (1) CHB1 (12)	SC01 (4) SC02 (3)
Family Ursidae			
Asiatic black bear (*Ursus thibetanus*)	15	CHB1 (9)	SC01 (4) SC02 (2)
Brown bear (*Ursus arctos*)	1	CHB1 (1)	
Family Procyonidae			
Northern raccoon (*Procyon lotor*)	2	D (1)	SC02 (1)
Family Ailuridae			
Red panda (*Ailurus fulgens*)	2	CHB1 (2)	
Order Primate	2 (2.7)	D (1) CHB1 (1)	
Family Lemuridae			
Ring-tailed lemur (*Lemur catta*)	1	CHB1 (1)	
Family Hylobatidae			
Northern white-cheeked gibbon (*Nomascus leucogenys*)	1	D (1)	
Total	22 (29.7)	D (2) CHB1 (13)	SC01 (4) SC02 (3)

**Fig. 1:**
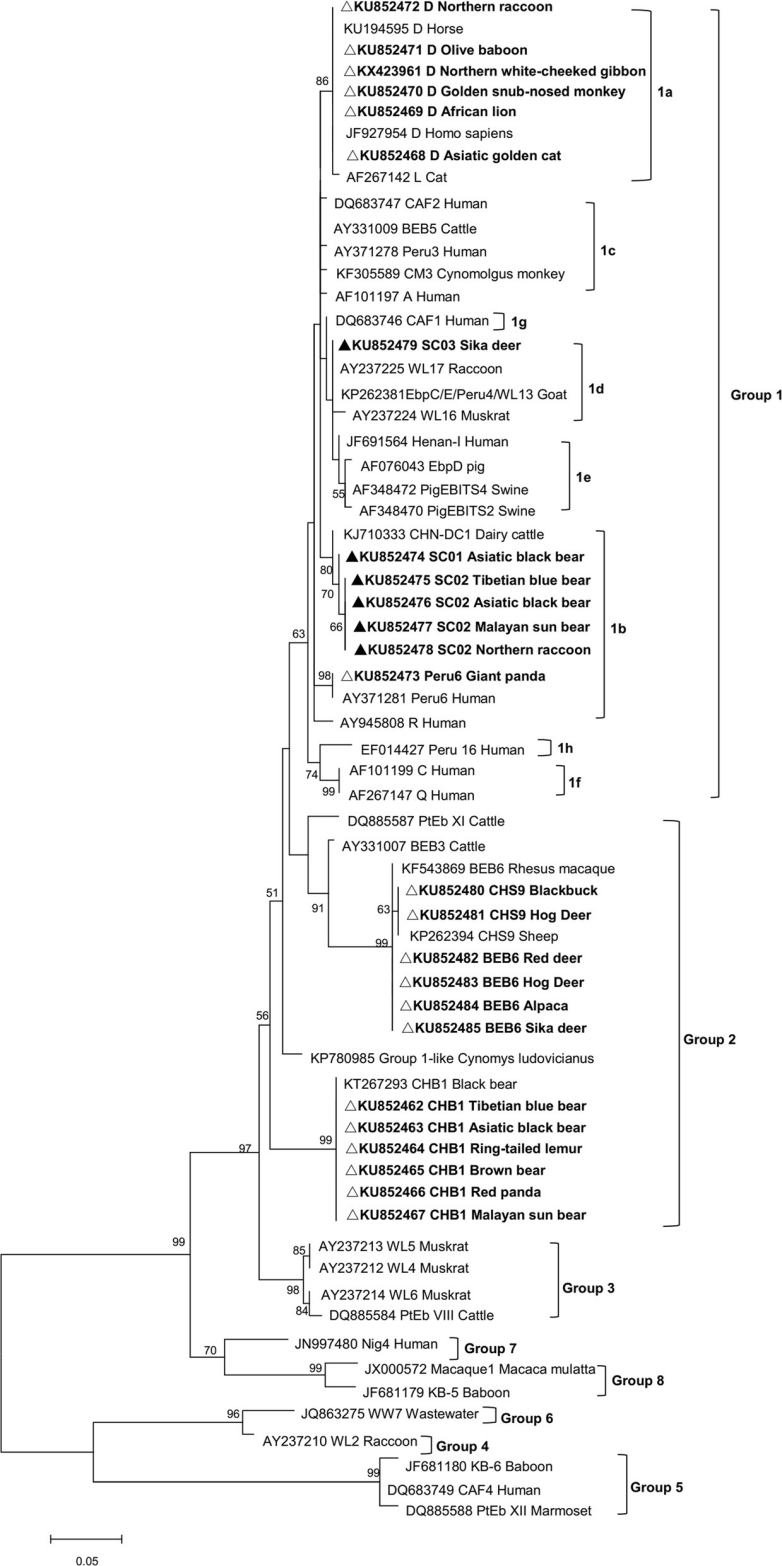
Phylogenetic relationships of ITS nucleotide sequences of the *Enterocytozoon bieneusi* genotypes identified in this study and other reported genotypes. The phylogeny was inferred by a maximum likelihood analysis. Bootstrap values were obtained using 1,000 pseudoreplicates and greater than > 50 % was shown on nodes. The genotypes in this study are marked by *empty* triangles and the novel genotypes are marked by *filled* triangles

Phylogenetic analyses based on ITS sequencing indicated that all representative isolates detected in this work belong to Group 1 or Group 2 (Fig. 1). Specifically, isolates with the known genotypes (D and Peru 6) and the three new genotypes (SC01, SC02 and SC03) fell into Group 1, while strains with genotypes CHB1, BEB6 or CHS9 were categorized as Group 2. Moreover, the genotype D strains identified in this study clustered into Subgroup 1a, while the Peru 6, SC01 and SC02 strains and the SCO3 strains were clustered into subgroups 1b and 1 day, respectively (Fig. 1).

ITS analysis of the three novel genotypes showed genetic variability. There was a three-nucleotide difference between the ITS of SC01 strains and that of genotype CM3 (KF305589). The ITS sequences of SC02 strains isolated from Tibetian blue bears, Asiatic black bears, sun bears and northern raccoons differed from that of the CHN-DC1 genotype (KJ710333) by two SNPs. Finally, the ITS sequences harbored by strains of the newly-identified genotype SC03 contained four SNPs relative to that of genotype EbpC (KP262381).

### MLST genotyping of *E. bieneusi* strains

In this study, 37, 33, 35 and 37 MS loci were successfully sequenced from the 43 ITS-positive specimens, respectively. For the MS1 locus, analysis of sequence polymorphisms, including trinucleotide TGC, TAA and TAC repeats, and single nucleotide polymorphisms (SNPs) revealed eight distinct types (Types I–VIII). The TA indels and SNPs present in MS3 indicated three types (Type I– III), and analysis of a 35 bp minisatellite repeat region (TTA TTT TTT CCA TTT TTC TTC TTC TAT TTC CTT TA) and two regions of indels (GGTA and TTT TTT TCT T) in MS4 yielded 11 distinct types (Type I–XI). Finally, the MS7 marker exhibited a trinucleotide TAA repeat and several SNPs, generating 15 types (Type I–XV). Altogether, 31 specimens yielded positive amplification of each of these four loci, forming 27 distinct MLGs (Table 4).

**Table 4:** Multi-locus sequence typing of *Enterocytozoon bieneusi* in wild animals

Code	Host	Multi-locus sequence genotype					
		ITS	MS1	MS3	MS4	MS7	MLGs
Order Carnivora							
Family Ursidae							
CDZ13	Asiatic black bear (*Ursus thibetanus*)	SC02^a^	Type VII	Type I	Type VI	Type XIII	MLG8
BFX01	Asiatic black bear (*Ursus thibetanus*)	CHB1	Type VII	Type I	Type I	Type VII	MLG14
BFX02	Asiatic black bear (*Ursus thibetanus*)	CHB1	Type VII	Type I	Type I	Type I	MLG15
BFX03	Asiatic black bear (*Ursus thibetanus*)	CHB1	Type VII	Type I	Type I	Type II	MLG16
BFX04	Asiatic black bear (*Ursus thibetanus*)	CHB1	Type VII	Type I	Type I	Type VI	MLG17
BFX05	Asiatic black bear (*Ursus thibetanus*)	CHB1	Type VII	Type I	Type I	Type VII	MLG14
BFX06	Asiatic black bear (*Ursus thibetanus*)	CHB1	Type VII	Type III	Type I	Type V	MLG18
BFX07	Asiatic black bear (*Ursus thibetanus*)	SC01^a^	Type VIII	Type II	Type VIII	Type XIII	MLG19
BFX08	Asiatic black bear (*Ursus thibetanus*)	CHB1	Type VIII	Type I	Type I	Type XIII	MLG20
BFX09	Asiatic black bear (*Ursus thibetanus*)	SC01^a^	Type VIII	Type II	Type VIII	Type XIII	MLG19
BFX10	Asiatic black bear (*Ursus thibetanus*)	SC02^a^	Type VII	Type II	Type I	Type XII	MLG21
BFX11	Asiatic black bear (*Ursus thibetanus*)	SC02^a^	Type VII	Type II	Type I	Type IV	MLG22
BFX12	Asiatic black bear (*Ursus thibetanus*)	SC01^a^	Type VIII	Type II	Type IX	Type XIII	MLG23
BFX13	Asiatic black bear (*Ursus thibetanus*)	CHB1	Type VII	Type I	Type VII	Type VII	MLG24
BFX14	Asiatic black bear (*Ursus thibetanus*)	SC01^a^	Type VIII	Type II	Type XI	Type XIII	MLG23
BFX15	Asiatic black bear (*Ursus thibetanus*)	CHB1	Type VII	Type I	Type I	Type IX	MLG25
CDZ21	Giant panda (*Ailuropoda melanoleuca*)	Peru 6	Type IV	ns	Type X	Type VIII	
CDZ03	Malayan sun bear (*Helarctos malayanus*)^b^	CHB1	Type VIII	Type I	Type V	Type II	MLG3
CDZ15	Malayan sun bear (*Helarctos malayanus*)^b^	SC02^a^	Type VIII	Type II	Type V	Type XIII	MLG10
BFX20	Brown bear (*Ursus arctos*)^b^	CHB1	Type VI	Type I	Type I	Type II	MLG27
CDZ05	Tibetian blue bear (*Ursus arctos pruinosus*)^b^	SC02^a^	Type VIII	Type II	Type II	Type XIII	MLG4
CDZ14	Tibetian blue bear (*Ursus arctos pruinosus*)^b^	CHB1	Type VII	Type I	Type I	Type III	MLG9
Family Ailuridae							
BFX21	Red panda (*Ailurus fulgens*)	CHB1	ns	ns	ns	Type XI	
BFX22	Red panda (*Ailurus fulgens*)	CHB1	ns	ns	ns	Type XIII	
Family Felidae							
CDZ04	African lion (*Panthera leo*)	D	Type VIII	ns	ns	ns	
CDZ16	African lion (*Panthera leo*)	D	Type VIII	Type I	Type I	ns	
CDZ01	Asiatic golden cat (*Catopuma temminckii*)^b^	D	Type VIII	Type I	Type IV	Type XIII	MLG1
Family Procyonidae							
BFX16	Northern raccoon (*Procyon lotor*)	D	ns	ns	ns	ns	
BFX17	Northern raccoon (*Procyon lotor*)	SC02^a^	ns	ns	ns	Type XIII	
Order Artiodactyla							
Family Cervidae							
CDZ02	Red deer (*Cervus elaphus*)	BEB6	Type VIII	Type II	Type IV	Type XIV	MLG2
CDZ07	Hog Deer (*Axis porcinus*)^b^	BEB6	ns	Type I	ns	ns	
CDZ08	Hog Deer (*Axis porcinus*)^b^	CHS9	Type VIII	Type I	Type IV	Type XV	MLG5
CDZ10	Hog Deer (*Axis porcinus*)^b^	BEB6	Type VIII	Type I	Type IV	ns	
CDZ11	Sika deer (*Cervus nippon*)	BEB6	Type III	Type II	Type I	Type VII	MLG6
CDZ12	Sika deer (Cervus nippon)	SC03^a^	Type VIII	Type I	Type IV	Type VII	MLG7
Family Camelidae							
CDZ09	Alpaca (*Lama pacos*)	BEB6	ns	ns	ns	ns	
Family Bovidae							
CDZ06	Blackbuck (*Antilope cervicapra*)^b^	CHS9	Type I	ns	Type IV	Type XV	
Order Primates							
Family Cercopithecidae							
CDZ17	Golden snub-nosed monkey (*Rhinopithecus roxellana*)	D	Type VIII	Type I	Type IV	Type VI	MLG11
CDZ18	Golden snub-nosed monkey (*Rhinopithecus roxellana*)	D	Type VIII	ns	ns	Type XIII	
CDZ19	Olive baboon (*Papio anubis*)	D	Type VIII	Type I	Type III	Type XIII	MLG12
CDZ20	Olive baboon (*Papio anubis*)	D	Type II	Type I	Type III	Type XIII	MLG13
Family Hylobatidae							
BFX18	Northern white-cheeked gibbon (*Nomascus leucogenys*)	D	Type V	Type III	Type IV	Type X	MLG26
Family Lemuridae							
BFX19	Ring-tailed lemur (*Lemur catta*)	CHB1	Type VII	Type I	Type I	Type II	MLG16

^a^Novel genotype identified in this study

^b^Animal species infected with *Enterocytozoon bieneusi* reported for the first time

*Abbreviation*: *ns*, not successfully sequenced or unsuccessful PCR amplification

## Discussion

The results of this study demonstrate the occurrence of *E. bieneusi* infections among wildlife housed in zoos in southwest China. The overall infection rates in Chengdu Zoo and Bifengxia Zoo were 10.6 % and 29.7 %, respectively, indicating that *E. bieneusi* is a particularly common pathogen at Bifengxia Zoo. In contrast, Li et al. [26] previously detected *E. bieneusi* in 15.8 % of wildlife at Zhengzhou Zoo. Animals of the order Carnivora exhibited infection rates of 4.0 % and 27.0 % at Chengdu and Bifengxia Zoo, respectively. Prior studies have reported a similar range of *E. bieneusi* infection rates among animals of this order in China, including 11 % in pandas, 5.8 % in cats, 6.7 % in dogs, 12.3–27.7 % in foxes and 10.5 % in raccoon dogs [15, 16, 27, 28]. The low prevalence of *E. bieneusi* infections observed among animals of the order Primates in Chengdu Zoo (2 %) was nearly identical to that detected at Bifengxia Zoo (2.7 %). Notably, these infection rates were markedly lower than those detected at other zoos (15.2 %–44.8 %) by Karim et al. [29]. Similarly, previous studies detected infection rates of 11.5 % among a group of primates comprised of 23 nonhuman primate (NHP) species (158/1,386 animals) [30] and of 28.2 % and 12.3 % among free-ranging rhesus monkeys and newly captured baboons in Kenya, respectively [13, 31]. The prevalence of *E. bieneusi* among animals of the order Artiodactyla was 4 % in Chengdu Zoo, which was similar to that observed in golden takins (4.7 %) in a prior study [1], but markedly lower than the average infection rate detected in reindeers (16.8 %) and in dairy cattle (24.3 %) [32, 33]. Notably, no such animals tested at Bifengxia Zoo were infected with *E. bieneusi*. The results of this investigation indicate that the occurrence of *E. bieneusi* varies between zoos and animal species.

ITS sequencing and phylogenetic analyses detected two known (D and Peru 6) and three novel *E. bieneusi* genotypes (SC01, SC02 and SC03) among Group 1 strains, which exhibit zoonotic potential, whereas Group 2 was comprised of the CHB1, BEB6 and CHS9 genotypes. In Chengdu Zoo, seven genotypes were detected, with the zoonotic D genotype being the most prevalent followed by Peru 6. Notably, genotype D was found in four animal species, suggesting cross-transmission between these animals. Indeed, previous reports have detected genotype D in humans and wildlife in various different countries [34–37]. Therefore, these findings indicate that zoonotic transmission to humans and between wildlife species may occur in Chengdu Zoo. In Bifengxia Zoo, four *E. bieneusi* genotypes were identified, with CHB1 being the predominant genotype; this genotype was especially common among black bears. Our results provide the first evidence of CHB1 infection among Malayan sun bears, red pandas, brown bears, Tibetian blue bears and ring-tailed lemurs. Additionally, a recently published study reported CHB1 infection in black bears [26]. The common existence of the CHB1 genotype among animals of the family Ursidae indicates that these animals may be more susceptible to infection by *E. bieneusi* than other species housed at this zoo.

In this study, *E. bieneusi* infection was detected in a total of 18 wildlife species. Of these, six had previously never been found to be infected with this organism, including Asiatic golden cats, Tibetian blue bears, Malayan sun bears, brown bears, blackbucks and hog deer. As such, our findings extend the known host-range for this parasite. These newly identified hosts belong to the order Artiodactyla or Carnivora, indicating that animals in these orders may be more susceptible to infection by *E. bieneusi*. Two Malayan sun bears and an alpaca were observed to be infected with CHB1 and BEB6, respectively. In contrast, genotype J was identified in Malayan sun bears and genotypes CHALT1 and J were detected in alpacas in Zhengzhou Zoo [26]. Similar to the results of a previous study, we detected genotype BEB6 in sika deer [38]; we also detected the newly identified genotype SC03 in these animals. Interestingly, the isolation of two novel genotypes from these deer, as well as the five novel genotypes identified by the study of Zhao et al. [38] suggest that genetic variability between deer-derived *E. bieneusi* may be common. While previous studies reported infections with genotypes D, Ebpc, WL1, WL2, WL3 and WL15 in raccoons [26, 39], we detected only genotypes D and SC02 in these animals. However, the presence of the novel SC02 genotype indicates that raccoons likely harbor strains of *E. bieneusi* that have yet to be characterized. Tian et al. [27] detected the I-like and EbpC genotypes in giant pandas and red pandas, respectively. In contrast, we detected the Peru 6 genotype in giant pandas and the CHB1 genotype in red pandas. Recently, several studies have examined the prevalence and types of *E. bieneusi* infections among NHP species. These studies demonstrated that NHP can be infected by a wide range of genotypes, including Type IV, D, Henan V, Peru8, PigEBITS7, EbpC, WL15, LW1d, Peru11, Peru7, BEB6, I, O, EbpA, Henan-IV, BEB4, PigEBITS5, EbpD, CS-1, CM1–CM18, Macaque 1, Macaque 2 and KB1–KB6 [29–31, 40]. Here, we further these findings by providing the first evidence that NHP can be also infected by genotype CHB1, and by demonstrating that northern white-cheeked gibbons can harbor genotype D.

MLST analyses involving the amplification and sequencing of housekeeping genes are widely used to study genetic profiles of pathogens with high resolution, sensitivity and specificity. Indeed, this method plays an important role in parasite research, including in studies of *Cryptosporidium* and *E. bieneusi*, and has been applied to the evaluation of *E. bieneusi* strains isolated from humans, pandas, golden takins, baboons and other NHP [1, 22, 27, 40–46]. We therefore utilized this approach to analyze 43 ITS-positive *E. bieneusi* wildlife-derived isolates. Our analyses indicated that these 43 strains were comprised of 27 distinct MLGs. Several Asiatic black bears at Bifengxia Zoo were infected with the same three MLGs (MLG14, MLG19 and MLG23), indicating likely transmission of *E. bieneusi* between these animals. Interestingly, despite belonging to distinct orders, both a ring-tailed lemur (Primates) and an Asiatic black bear (Carnivora) were infected with MLG16; however, it is unclear which animal was the source of the infection. To our surprise, the same MLG was never detected within two animals of the same species at Chengdu Zoo. Indeed, 12 distinct MLGs were detected among Asiatic black bears. Likewise, other species were infected with multiple different MLGs. These findings indicate that there is a significant level of genetic variability among *E. bieneusi* strains.

## Conclusions

The results of our study describe the prevalence of *E. bieneusi* infections among captive wildlife in zoos in southwest China. Furthermore, they provide the first evidence of *E. bieneusi* infections in Asian golden cats, Tibetian blue bears, blackbucks, hog deer, Malayan sun bears and brown bears, thereby expanding the recognized host-range of this organism. The detection of zoonotic genotypes among various animals highlights the potential for zoonotic transmission to humans. Thus, methods for controlling this transmission are needed. Our novel *E. bieneusi* sequencing data will facilitate future molecular epidemiology research. However, further multi-locus genotyping analyses, involving a larger number of isolates from humans and wildlife, are needed to better assess the zoonotic potential and transmission dynamics of *E. bieneusi*.

## Abbreviations

ITS, internal transcribed spacer; MLST, Multilocus sequence typing; MLGs, Multilocus genotyping; SNPs, single nucleotide polymorphisms

## Additional files

Additional file 1: Table S1. List of mammals in Chengdu Zoo examined in the present study. Common name, scientific name, number of specimens tested and number of samples positive for *Enterocytozoon bieneusi*, genotypes of *Enterocytozoon bieneusi* are provided. (XLSX 12 kb) Additional file 2: Table S2. List of mammals in Bifengxia Zoo examined in the present study. Common name, scientific name, number of specimens tested and number of samples positive for *Enterocytozoon bieneusi*, genotypes of *Enterocytozoon bieneusi * are provided. (XLSX 10 kb)
